# Development and validation of a deep learning survival model for cervical adenocarcinoma patients

**DOI:** 10.1186/s12859-023-05239-7

**Published:** 2023-04-13

**Authors:** Ruowen Li, Wenjie Qu, Qingqing Liu, Yilin Tan, Wenjing Zhang, Yiping Hao, Nan Jiang, Zhonghao Mao, Jinwen Ye, Jun Jiao, Qun Gao, Baoxia Cui, Taotao Dong

**Affiliations:** 1 Cheeloo College of Medicine, Shandong University, No. 44 Wenhua West Road, Lixia District, Jinan, 250012 Shandong Province China; 2 Department of Obstetrics and Gynecology, Qilu Hospital of Shandong University, No. 107, Wenhua West Road, Jinan, 250012 Shandong Province China; 3 Department of Obstetrics and Gynaecology, Qilu Hospital (Qingdao), Cheeloo College of Medicine, Shandong University, Qingdao, China; 4 Department of Obstetrics and Gynecology, Affiliated Hospital of Qingdao University, No. 16, Jiangsu Road, Shinan District, Qingdao, 266555 Shandong Province China

**Keywords:** Adenocarcinoma of cervix, Survival prediction, Deep learning, SEER database

## Abstract

**Background:**

The aim was to develop a personalized survival prediction deep learning model for cervical adenocarcinoma patients and process personalized survival prediction.

**Methods:**

A total of 2501 cervical adenocarcinoma patients from the surveillance, epidemiology and end results database and 220 patients from Qilu hospital were enrolled in this study. We created our deep learning (DL) model to manipulate the data and evaluated its performance against four other competitive models. We tried to demonstrate a new grouping system oriented by survival outcomes and process personalized survival prediction by using our DL model.

**Results:**

The DL model reached 0.878 c-index and 0.09 Brier score in the test set, which was better than the other four models. In the external test set, our model achieved a 0.80 c-index and 0.13 Brier score. Thus, we developed prognosis-oriented risk grouping for patients according to risk scores computed by our DL model. Notable differences among groupings were observed. In addition, a personalized survival prediction system based on our risk-scoring grouping was developed.

**Conclusions:**

We developed a deep neural network model for cervical adenocarcinoma patients. The performance of this model proved to be superior to other models. The results of external validation supported the possibility that the model can be used in clinical work. Finally, our survival grouping and personalized prediction system provided more accurate prognostic information for patients than traditional FIGO stages.

**Supplementary Information:**

The online version contains supplementary material available at 10.1186/s12859-023-05239-7.

## Background

Cervical cancer is the fourth most common cancer in females, causing 604,127 new cases in 2020 worldwide [1]. Though adenocarcinoma only accounts for 10–25% of all cervical cancer cases, its greater propensity to metastasis leads to poor prognosis [2, 3]. According to The National Comprehensive Cancer Network (NCCN) guidelines, the primary treatment of early-stage cervical cancer is surgery. But the current guidelines don’t distinguish different strategies for squamous cell carcinoma (SCC) and adenocarcinoma (AC). Several factors have been recognized as associated with survival outcomes after surgery. The International Federation of Gynecology and Obstetrics (FIGO) stage is the most investigated prognostic factor for cervical AC with five-year overall survival (OS) being 79% in stage I and 37% in stage II [2, 4]. Besides that, lymph node status, tumor size, tumor grade, depth of cervical invasion, patients' age, lymphovascular-space involvement, and parametrial involvement are also identified as prognostic factors [5–7].

Most prognostication studies for cervical AC were developed with multivariate analysis, Cox proportional hazards (CPH) regression analysis, and the Kaplan–Meier (K–M) survival curve model [8–10]. However, these traditional methods have been proven to be less accurate in the survival prediction of some cancers than those new models like the linear multi-task (LMT) model, random survival forest (RSF) model, support vector machine (SVM) model and deep learning (DL) model [11, 12]. The DL model, as a newly emerging model, allows the automatic discovery of the representations with the use of fully connected layers in the network and can analyze the nonlinear correlations that are more common in the real world [13]. Until now, no study has been carried out for cervical AC patients to develop a new survival prediction DL model and to compare the predictive accuracy of different models.

However, a large number of cases are needed for the DL model to output more accurate prediction results. Due to the relatively low incidence and poor prognosis of cervical AC, large cohort studies in the real world are difficult to carry out. The surveillance, epidemiology, and end results (SEER) database provides a new choice for researchers. The SEER database is a population-based data source covering approximately 34.65% of the U.S. population [14]. Clinical data and follow-up information for all tumor patients have been collected since 1973. The huge number of medical records has enabled it to provide information for the survival analysis of a variety of cancers [15–17] and to satisfy demands for these machine learning models [18, 19]. Besides, another challenge for developing DL systems in survival prediction is clinical validation. The use of a single dataset for model development and validation leads to the risk of overfitting which is a common prediction error in machine learning [20]. Thus, validation of the DL model in external datasets, especially real word clinical records, is necessary.

In this study, we aimed to develop a survival prediction DL model for cervical AC patients who have had surgeries. To verify the reliability of the new model, real word data from a medical center in China was also included as an external-test set. We made a systematic comparison of different models, including the CPH model, LMT model, RSF model, SVM model, and DL model. We also developed risk grouping based on survival prediction and a new personalized survival prediction system based on the DL model.

## Materials and methods

### Data collection in SEER database

The SEER database had 133 usable variables including cancer stage at the time of diagnosis and patient survival data. In this study, we used the “International Classification of Disease for Oncology, Third Edition (ICD-O-3)” for the selection of primary cervical cancer patients diagnosed from 1973 to 2014. The selection codes for ICD-O-3 were C53.0 (Endocervix), C53.1 (Exocervix), C53.8 (Overlapping lesion of cervix uteri), and C53.9 (Cervix uteri). The selection codes for histology are adenomas and adenocarcinomas (the description of the “histo3v” code is 8140 and 8389). We kept cervical AC patients who have had surgery. Cases with multiple tumors were excluded and the final sample size was 2501 (Table 1). For the missing values, we filled up them with the mean of each variable when building the model. Detailed information can be found in the Additional file 1.

**Table 1:** Patient demographic characteristics in SEER database

	Characteristics	No.	%
Age, years (N = 2501)	Mean	44	
	SD	12.7	
Race (N = 2480)	White	2049	82.62
	Black	144	5.81
	Asian	287	11.57
Marital status (N = 2422)	Single	478	19.74
	Married	1504	62.10
	Separated	37	1.53
	Divorced	267	11.02
	Widowed	136	5.62
Stage (N = 2465)	IA	838	34.00
	IB	1372	55.66
	IIA	104	4.22
	IIB	151	6.13
Lymph node metastasis (N = 2395)	No metastasis	2190	91.44
	Regional lymph node	179	7.47
	Aortic/distant lymph node	26	1.09
Positive lymph node numbers (N = 1814)	0	1616	89.08
	1	88	4.85
	2	43	2.37
	3	26	1.43
	4	10	0.55
	> = 5	31	1.72
Resected lymph node numbers (N = 2311)	Mean	14.3	
	SD	13.6	
Tumor diameter, mm (N = 1359)	Mean	23.7	
	SD	40.6	
Depth of invasion (N = 1359)	Inner 1/3	770	56.66
	Middle 1/3	250	18.40
	Outer 1/3	339	24.94
Differentiation (N = 1942)	Low	435	22.40
	Moderate	752	38.72
	High	755	38.88
Surgery (N = 955)	Local excision	75	7.85
	TH	49	5.13
	TH + LND	65	6.81
	TH + BSO	127	13.3
	TH + BSO + LND	639	66.9

Since the SEER dataset utilized publicly available desensitized data, data from the database did not need approval from the institutional review board (IRB) or informed consent from patients.

### Data preparation in SEER database

**Fig. 1:**
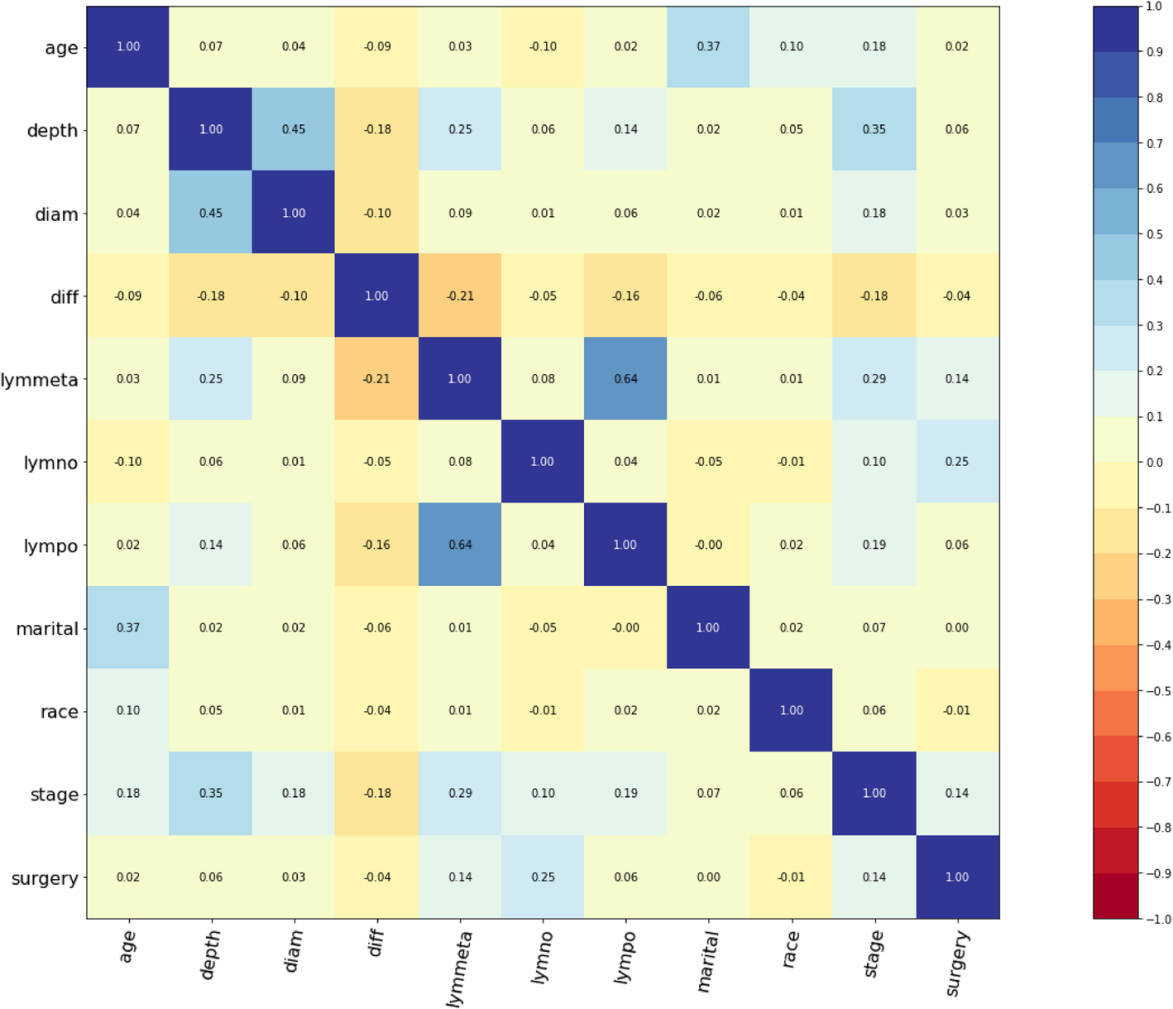
Correlation matrix of 11 selected variables. Values in this figure indicated the correlation coefficient of two corresponding variables. The color indicated the strength of the correlation. Depth: depth of invasion. Diam: tumor diameter. Diff: differentiation. Lymmeta: lymph node metastasis. Lymon: resected lymph node numbers. Lympo: positive lymph node numbers

According to the clinical definition of cervical adenocarcinoma and the year of data entry, we selected variables to be analyzed. Then, we excluded those duplicated variables using correlation matrix analyses. According to the clinical definition of cervical adenocarcinoma, we selected some variables to be analyzed. Then, we excluded those duplicated variables using correlation matrix analyses, and setting correlation coefficient threshold: 0.7 (Fig. 1). Thus, a total of 11 variables were selected for further analyses among 133 original variables in the SEER database, including age, race, marital status, stage, lymph node metastasis, positive lymph node numbers, resected lymph node numbers, tumor diameter, depth of invasion, differentiation and surgery.

We use whole numbers to encoded these categorical variables, such as variable differentiation, we encoded low, moderate and high differentiation as 0,1,2 respectively. Stages were defined from the farthest extension of the tumor and whether lymph nodes were involved. The SEER catalog is named “rename eod10_ex”. Depth of invasion referred to extent of tumor invasion to the cervix and was defined according to “eod10_sz” and “CS Tumor Size/Ext Eval (2004 +)” in the SEER catalog. Depth of invasion was defined as a categorical variable indicating depth less than 1/3, depth between 1/3 and 2/3, and depth deeper than 2/3. Lymph node status was clearly described in the database according to the “eod10_nd”, “eod10_pn”, and “eod10_ne” catalog, consisting of lymph node metastasis, positive lymph node numbers and resected lymph node numbers, lymph node metastases were defined as categorical variables, indicating no metastases, pelvic lymph node metastases, or paraaortic lymph node metastases, the number of positive lymph nodes and the number of dissected lymph nodes were defined as continuous variables. In the SEER database, several methods were introduced to define race. In this study, we classified race into White, Black, and Asian as a categorical variable according to the catalog “rac_recy”. Marital status was defined as single, married, separated, divorced, and windowed according to catalog “rename mar_stat”. Differentiation was defined as a categorical variable indicating low, moderate, and high according to catalog “grade”. Surgery was also a categorical variable consisting of local excision, total hysterectomy (TH), total hysterectomy and lymph node dissection (TH + LND), total hysterectomy, and bilateral salpingo-oophorectomy (TH + BSO), and total hysterectomy and bilateral salpingo-oophorectomy plus lymph node dissection (TH + BSO + LND) according to catalog “ss_surg”. In addition, another two continuous variables were age and tumor diameter.

To make attribute values of variables lie numerically in the same scale, and have the same importance, before passing the input variables through the model, we preprocess our data by min–max scale using the “minmax_scaling” package [21] in python.

### Patient characteristics in the SEER database

A total of 2501 corpus adenocarcinoma patients registered from 1973 to 2014 in the SEER database were enrolled in this study. According to correlation analyses, 11 variables of these patients were involved for analysis. The selected patients were split into a training set (n = 1501, 60%), validation set (n = 500, 20%) and testing set (n = 500, 20%).

The patient demographic characteristics are shown in Table 1. A total of 2049 cases were White (82.62%), 144 were Black (5.81%), and 275 were Asian (11.09%). A total of 478 cases were single (19.74%), 1504 were married (62.10%), 37 were separated, 267 were divorced (11.02%) and 136 were widowed (5.62%). 435 cases were poorly differentiated (22.40%), 752 were moderately differentiated (38.72%) and 755 were highly differentiated (38.88%). A total of 2190 patients had localized tumors (91.44%), 179 patients extended to regional lymph nodes (7.47%), and 26 patients extended to distance lymph nodes (1.09%). A total of 838 cases were stage IA (34.00%), 1372 were stage IB (55.66%), 104 were stage IIA (4.22%) and 151 were stage IIB (6.13%). 75 patients underwent local excision surgery (7.85%) and 639 underwent TH + BSO + LND (66.9%).

### Data in the external-test set

Cases in the external-test set were retrospectively collected at Qilu Hospital Shandong, China. Data were collected through medical records and annual telephone follow-ups. The median follow-up time was 48.4 months. Informed consent from the patients was exempt because of the retrospective nature of the study. The study was approved by the hospital’s ethics committees.

We included patients who underwent surgery in Qilu Hospital from August 2005 to March 2021 and were pathologically diagnosed with cervical AC. Patients who refused follow-up were excluded. We also excluded patients whose first operation was not carried out in Qilu Hospital and patients with multiple tumors. Finally, the number of cases included in the external-test set is 220 (Table 2). Clinical data including age, race, marital status, stage, lymph node metastasis, positive lymph node numbers, resected lymph node numbers, tumor diameter, depth of invasion, differentiation, and surgery were analyzed. Detailed information can be found in the Additional file 2.

**Table 2:** Patient demographic characteristics in the external test set

	Characteristics	No.	%
Age, years (N = 219)	Mean	46	
	SD	9.53	
Race(N = 220)	Asian	220	100.00
Marital status (N = 219)	Single	0	0
	Married	208	94.98
	Divorced	9	4.11
	Widowed	2	0.91
Stage (N = 158)	IA	15	9.49
	IB	137	86.71
	IIA	6	3.80
	IIB	0	0
Lymph node metastasis (N = 219)	No metastasis	193	88.13
	Metastasis	26	11.87
Positive lymph node numbers (N = 219)	0	177	80.82
	1	16	7.31
	2	10	4.57
	3	6	2.74
	4	4	1.83
	> = 5	6	2.74
Resected lymph node numbers (N = 219)	Mean	21	
	SD	7.27	
Tumor diameter, mm (N = 219)	Mean	28.4	
	SD	1.58	
Depth of invasion (N = 219)	Inner 1/3	103	47.03
	Middle 1/3	21	9.60
	Outer 1/3	95	43.37
Differentiation (N = 168)	Low	25	14.88
	Middle	76	45.24
	High	67	39.88
Surgery (N = 219)	Local excision	9	4.11
	TH	3	1.37
	TH + LND	2	0.91
	TH + BSO + LND	131	59.82
	RTH + BSO + LND	74	33.79

### DL model building and evaluation

The original multitask logistic regression (N-MTLR) model developed by Chun-Nam Yu [22] was adopted as a basis for our model. Our model was developed on the PyTorch framework [23]. Scikit-learn [21] and pandas packages [24] were also involved in the data processing.

The structure of the final deep learning network involved 6 fully-connected layers, each layer had 100 neurons. The grid search method was used for selecting optimal hyperparameters. Optimal hyperparameters were as follows: weight initialization method = glorot_uniform, optimizer = “Adam” [25], learning rate = 1e−4, l2 regularization = 1e−4, l2 smooth = 1e−2, dropout rate = 0.3, number of iterations = 3000. The ranges of each of the hyperparameters as: the number of neuron layers [2, 10]; the hidden number of neurons in each layer [2, 300]; learning rate [10e−6, 1]; l2 regularization [10e−4, 10e−2]; l2 smooth [10e−4, 10e−2]; dropout rate [0, 1]. To prevent the potential overfitting of machine learning model, We conducted additional assessments using the testing set.

Hyperparameters for CPH model, LMT model, RSF model, and SVM model were as follows: In CPH model, weight initialization method = glorot_uniform, l2 regularization = 1e−2, learning rate = 1e−4, topology error check = 1e−4. In LMT model, final model involved 4 hidden neuron layers, each hidden layer had 50 neurons, activation function is ReLU, weight initialization method = glorot_uniform, optimizer = “Adam” [25], learning rate = 1e−3, l2 regularization = 1e−2, l2 smooth = 1e−2, dropout = 0.2. In RSF model, number of trees = 200, maximum features = log2, maximum depth = 2, minimum node size = 5. In SVM model, kernel = Gaussian, scale = 0.25, weight initialization method = glorot_uniform, bias = True, learning rate = 1e−3, topology error check = 1e−3, l2 regularization = 1e−3.

Data from the SEER database were split into the training set, validation set, and testing set. The testing set and QL set were independently applied to evaluate the performance of our model. We used the concordance index (c-index) and the integrated Brier scores (IBS) to compare the performances of different models.

### Statistical analyses

Overall survival (OS) was the main indicator for survival outcome analyses and prediction. K–M curve and receiver operating characteristic (ROC) curve were performed for patients staged with the traditional staging system and new risk grouping system. The area under the curve (AUC) was also calculated to compare the prognosis prediction ability of the two staging methods. Finally, personalized survival curves were also plotted for randomly selected patients from the testing set. A z-score test [26] was constructed to statistically compare the C-index and AUC between the two models, the results were considered significant if the *P* value < 0.05. These analyses were conducted using R version 3.0 (R Foundation for Statistical Computing, Vienna, Austria). Besides, we used STATA software (version 13) for parts of the statistical analyses.

## Results

### Performance of DL model

**Fig. 2:**
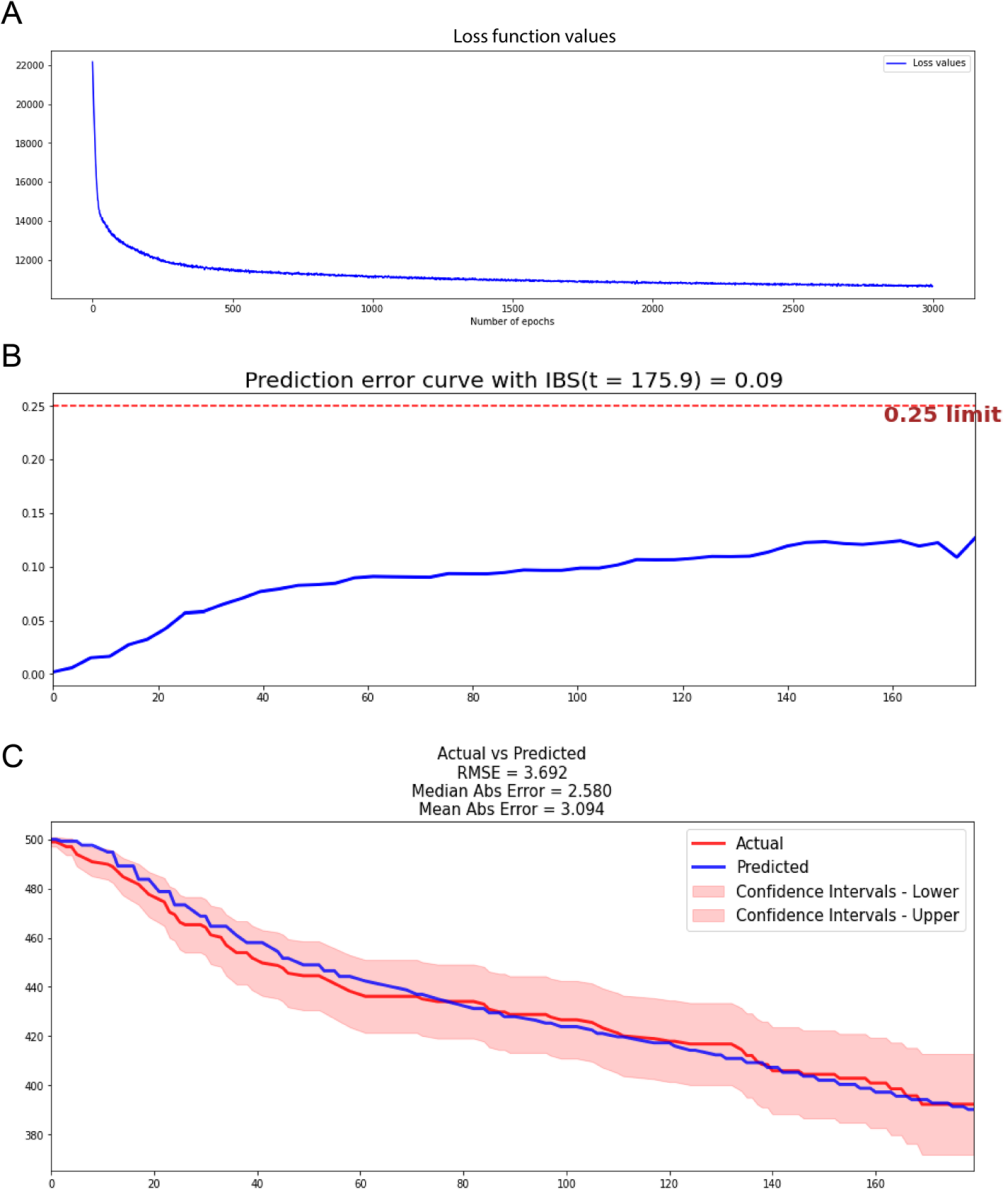
Performance of our DL model in the test set. **A** After 3000 iterations, the loss value decreased from 22,000 to 11,000. **B** The IBS of our DL model in the test set is 0.09. **C** Calibration survival curves of the testing set according to the DL model

The structure of the final deep learning network involved 6 neuron layers, each layer had 100 neurons. When iterations at 3000 the loss values curve tended to flatten (Fig. 2A).

To prevent the potential overfitting of machine learning model, We conducted additional assessments using the testing set. Finally, our model reached a c-index of 0.878 and an IBS of 0.09 in the testing set (Fig. 2B). In addition, Calibration curves showed that nearly all regions of the predicted survival curves were plotted within confidence intervals (Fig. 2C). 2.580 of the median absolute error (AE) and 3.094 of the mean AE were achieved in each time interval in testing set (Fig. 2C).

### Comparison of different models

**Fig. 3:**
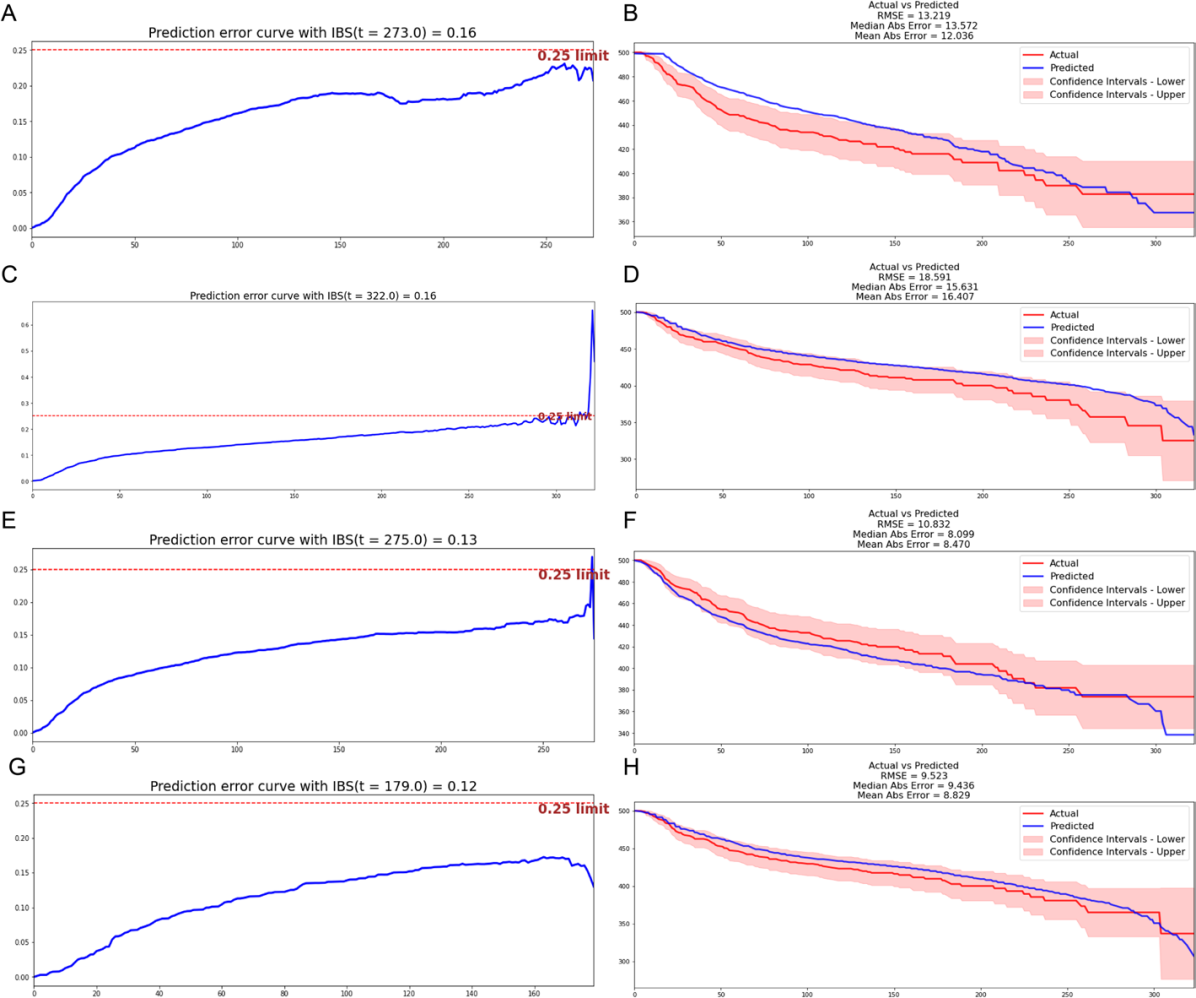
performance of other models including CPH model, LMT model, RSF model, and SVM model. **A** The IBS of the CPH model in the test set is 0.16. **B** Survival curves of the testing set according to the CPH model. **C** The IBS of the LMT model in the test set is 0.16. **D** Survival curves of the testing set according to the LMT model. **E** The IBS of the RSF model in the test set is 0.13. **F** Survival curves of the testing set according to the RSF model. **G** The IBS of the SVM model in the test set is 0.12. **H** Survival curves of the testing set according to the SVM model

We built the CPH model, LMT model, RSF model, and SVM model using the same data set from the SEER database. C-index and IBS were calculated, and actual and predicted survival curves were drawn for all models (Fig. 3). The CPH model reached 0.715 for the C-index, 0.16 for IBS, 13.572 for median AE, and 12.036 for mean AE (Fig. 3A, B). The LMT model reached 0.702 for the C-index, 0.16 for IBS, 15.631 for median AE, and 16.407 for mean AE. The predicted curve deviated from the confidence intervals (Fig. 3C, D). RSF model reached 0.737 for the C-index, 0.13 for IBS, 8.099 for median AE, and 8.470 for mean AE (Fig. 3E, F). The SVM model reached 0.693 for the C-index, 0.12 for IBS, 9.436 for median AE, and 8.829 for mean AE (Fig. 3G, H).

### DL model in the external test set

**Fig. 4:**
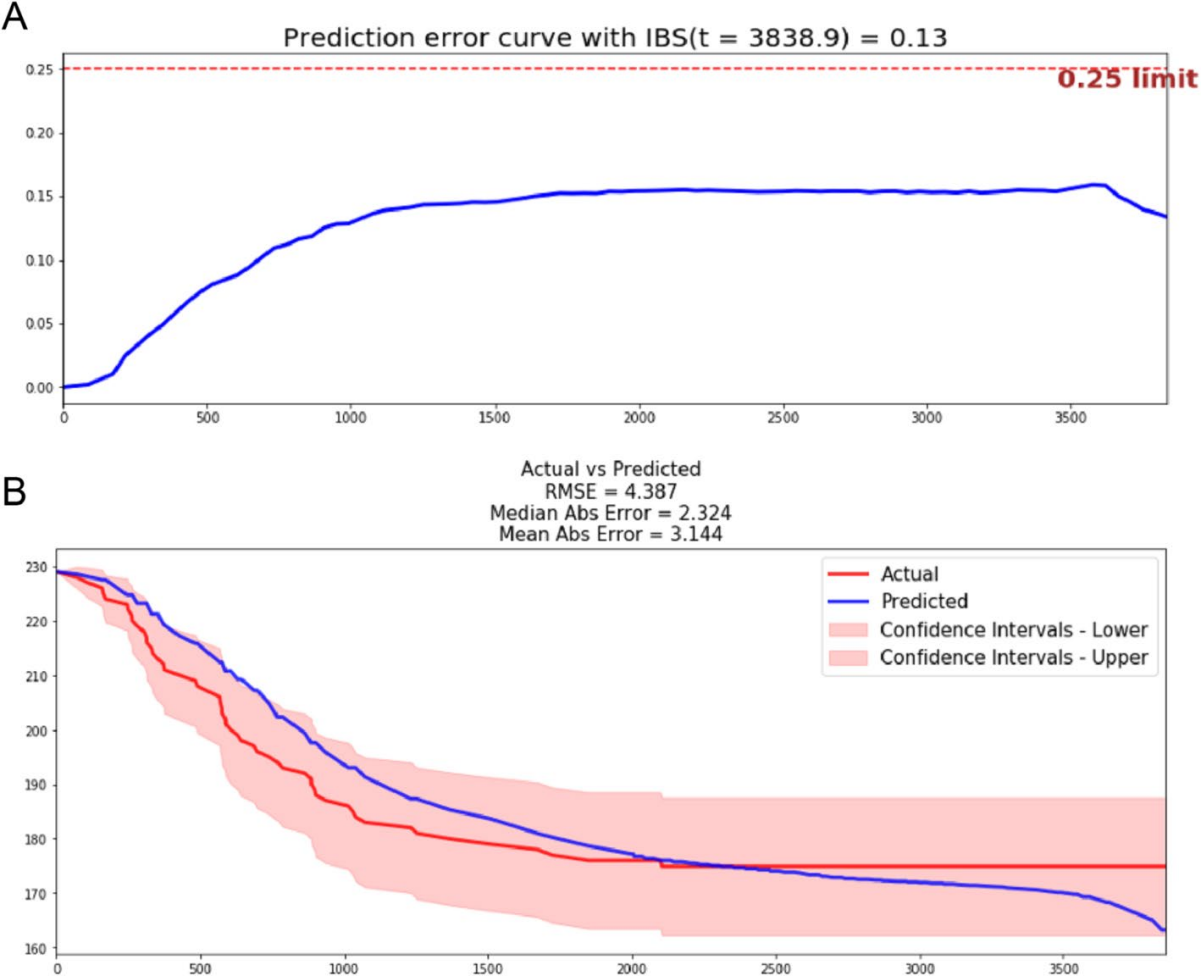
Validation of our DL model in the external test set. **A** The IBS of our DL model in the external-test set is 0.13. **B** Predicted survival curves according to the DL model located within confidence intervals

Finally, our model reached a c-index of 0.80 and an IBS of 0.13 in the external test set (Fig. 4A). Calibration curves showed the predicted survival curve located within confidence intervals. 2.324 of the median AE and 3.144 of the mean AE were achieved in each time interval (Fig. 4B).

### Prognosis-oriented risk grouping

**Fig. 5:**
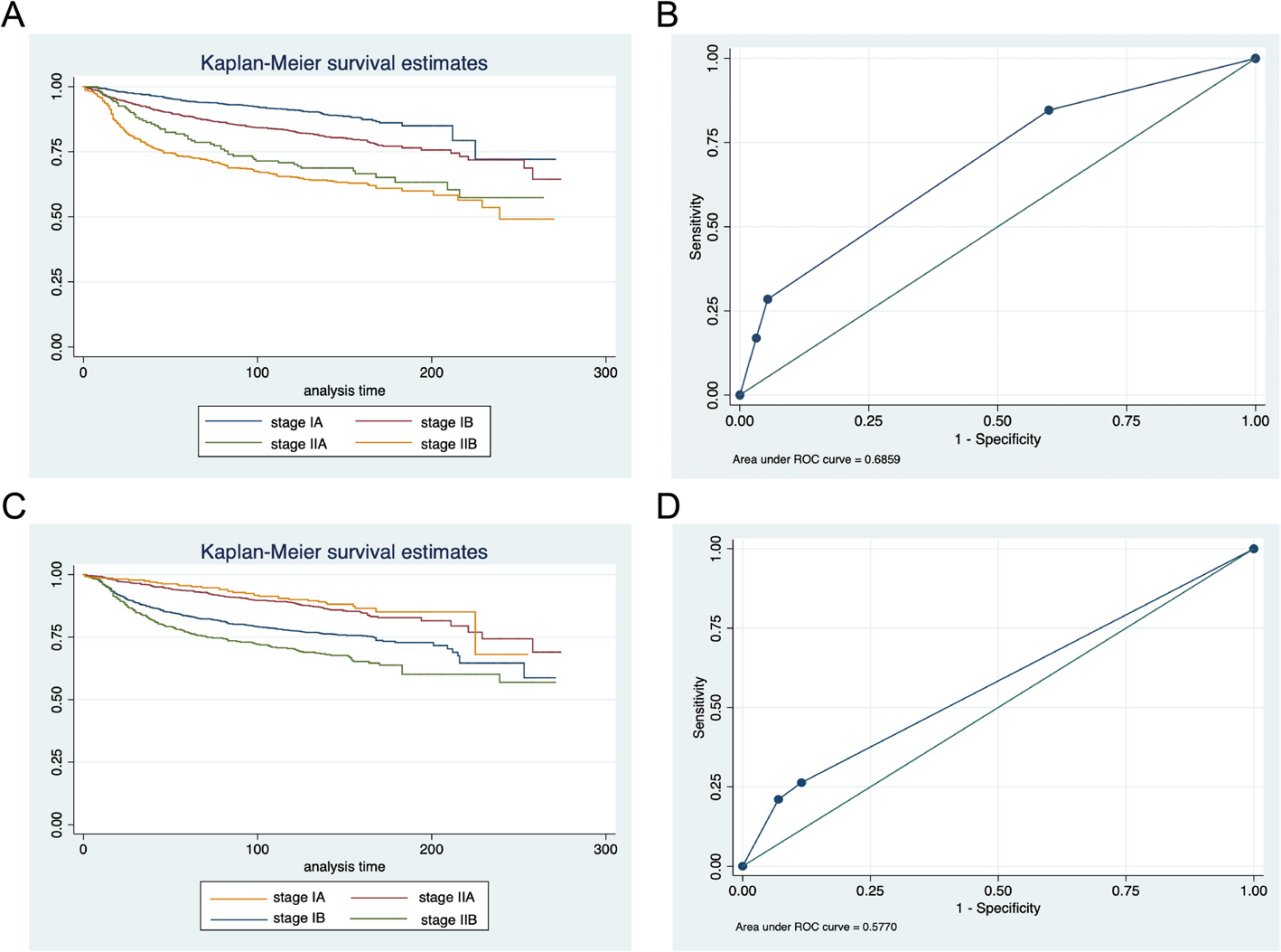
K–M curves and ROC curves of patients from the SEER database (**A**, **B**) and Qilu Hospital (**C**, **D**) according to the conventional staging system

K–M curves and ROC curves were plotted for patients from the SEER database and Qilu Hospital according to the conventional staging system (Fig. 5A–D). In the SEER database, mortality for stage II, III, and IV patients increased 2.21-, 6.35- and 7.28-fold relative to the stage I patients (95%CI 2.02–8.08, *P* < 0.0001). In the Qilu dataset, mortality for stage II and III patients increased 0.87- and 3.98-fold relative to the stage I patients (*P* > 0.05). The AUCs were 0.6859 and 0.5770 separately. The difference in survival between stages was inapparent.

**Fig. 6:**
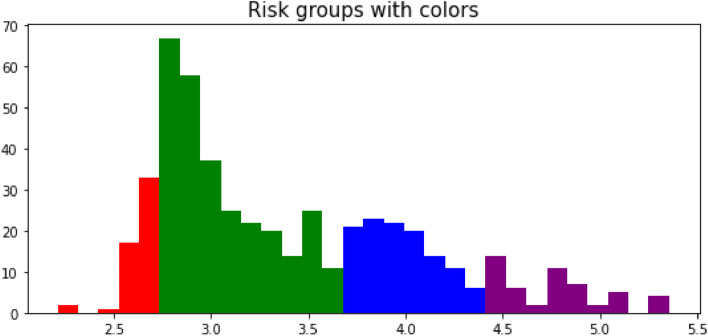
Four risk groups were divided according to a prognosis risk score calculated by our DL model

Risk factors for patients in the testing set and external test set were computed by our DSL model. According to their risk scores, patients were divided into four staging groups (Fig. 6). Patients with a score of 0–2.7 were classified in risk group I and marked in red color, patients with a score of 2.7–3.7 in risk group II and green color, patients with 3.7–4.4 scores in risk group III and blue color, patients with 4.4–5.5 score in risk group IV and purple color.

**Fig. 7:**
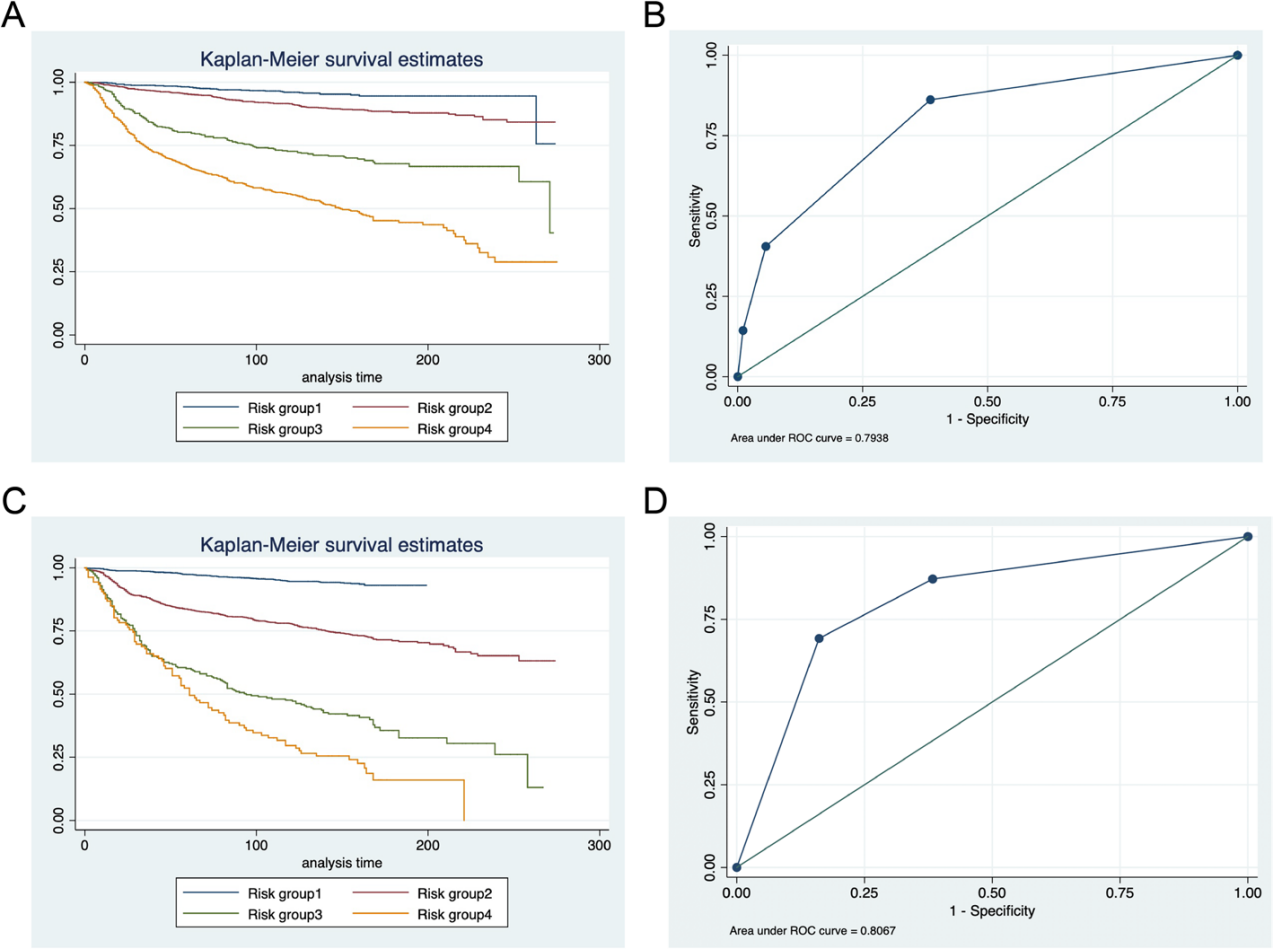
K–M curves and ROC curves of patients from the SEER database (**A**, **B**) and Qilu Hospital (**C**, **D**) according to the new prognosis-oriented grouping system

K–M curves and ROC curves were plotted for patients from the testing set and external test set according to our risk grouping system (Fig. 7A–D). In the test set, mortality for group II, III, and IV patients increased 2.19-, 7.09-, and 14.40-fold relative to the group I patients (95%CI 4.83–10.40, *P* < 0.0001). In the external test set, mortality for group II, III, and IV patients increased 4.84-, 14.56-, and 21.88-fold relative to the group I patients (95%CI 4.83–10.40, *P* < 0.0001). The AUROC was 0.7938 for the testing set and 0.8067 for the external test set.

### Personalized survival prediction using the DL model

**Fig. 8:**
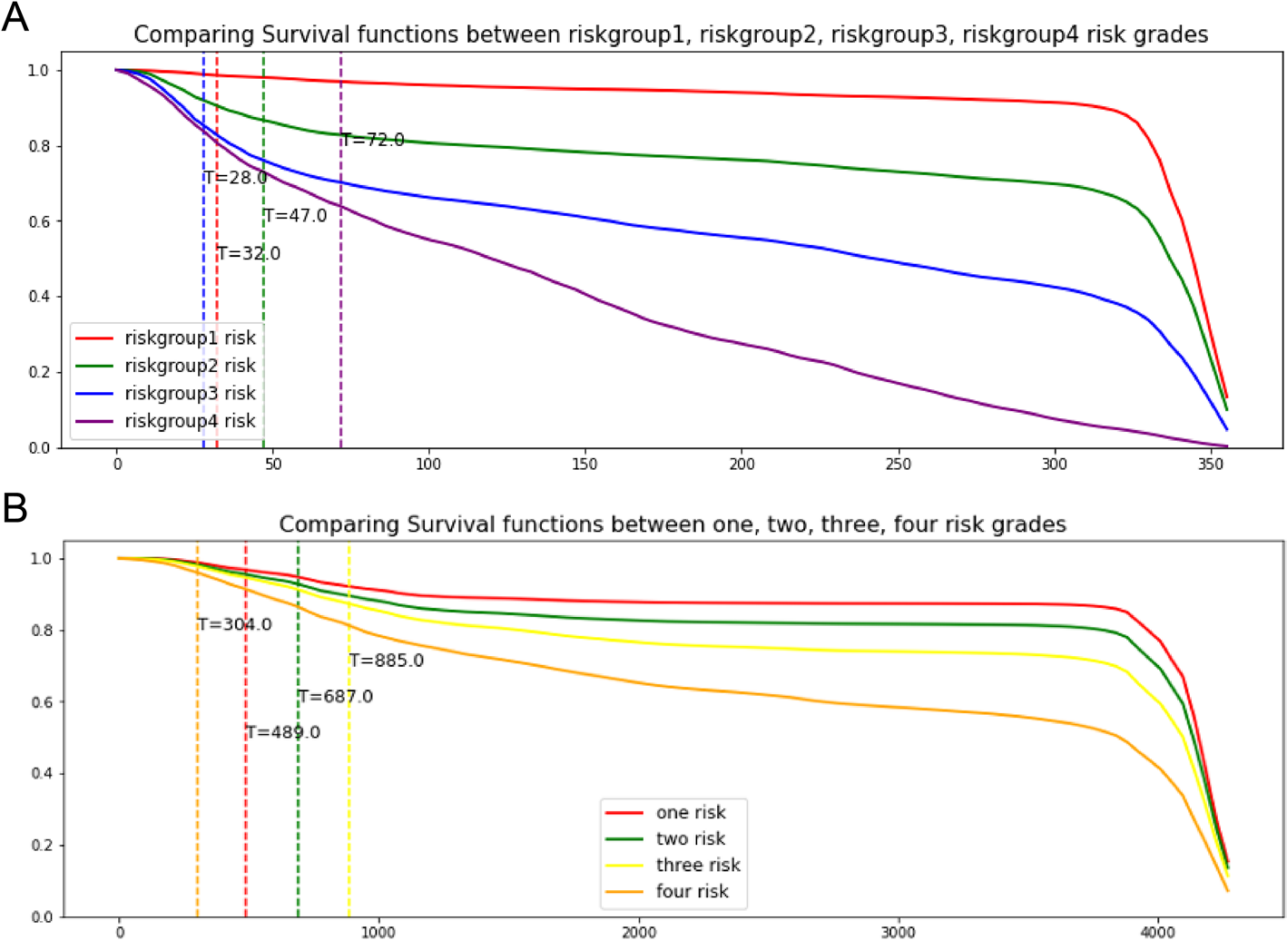
Personalized survival prediction using the DL model showed notable differences among patients in both test set (**A**) and external test set (**B**)

Then, we tried to process personalized survival prediction using our new model. A survival curve was drawn according to one single patient. To verify the accuracy of the personalized survival prediction, we painted survival curves for four patients who were randomly selected from each group of our risk grouping system. Notable differences among patients were observed in both the test set and the external test set (Fig. 8A, B).

## Discussion

Adenocarcinoma of the cervix is known as a relatively worse prognosis than squamous cell carcinoma. In this study, we established a deep learning model to predict survival outcomes for adenocarcinoma patients. To our knowledge, this is the first prognostication study for cervical adenocarcinoma patients applying a deep learning method.

In this study, we demonstrated that the new model had a good performance with a c-index of 0.80 and an IBS of 0.13 in the external test set. Besides, the accuracy of prediction supported by five different models was carefully compared and analyzed. In the test set, our model reached a c-index of 0.878 which was higher than that in the other four models, and IBS of 0.009 which was lower than that in the other four models. According to survival calibration curves, the predicted survival curve of our DL model almost coincided with the actual curve, while that of the LMT and SVM models deviated from the confidence intervals. Though survival calibration curves of RSF and CPH also didn’t visually deviate, the relatively low c-index and high IBS prevented them from being considered better models. All these data supportted the conclusion that the DL model was the most capable to complete survival analysis and provided the most accurate results. It was worth noting that the predicted survival curve drawn by the DL model in the external test set was completely located within the confidence intervals. A relatively small sample size in the external test set might contribute to this result.

Besides, we also demonstrated a new grouping system oriented by survival outcomes. The K–M curve drawn according to our new grouping system showed a more significant difference in survival rate. The AUCs were also higher with 0.7938 versus 0.6859 in the test set and 0.8067 versus 0.5770 in the Qilu dataset. There was no doubt that the traditional staging system was of comprehensive significance in guiding treatment and prognosis. However, when considering the survival outcomes, our grouping system had better prediction ability than the traditional staging system. Finally, in pursuit of more accurate survival prediction, we developed a personalized prediction system that could draw a predicted survival curve for a single patient. This personalized system showed strong performance on a validation set of randomly selected patients.

Previous studies have explored the ability of the CPH model to investigate prognostic factors of cervical adenocarcinoma. However, the conventional model, like the CPH model, could only deal with simple linear relationships between a prognostic factor and survival outcome. Complex nonlinear relationships existed among different factors which work together to influence the outcome. Thus, our DL model showed better performance by making up for this defect, which was consistent with results in other cancers [27, 28]. In addition, past works have never concentrated on the staging system and prognosis-related subgroups in cervical adenocarcinoma. The personalized prediction system was also unprecedented. Our work would provide new ways to predict survival for cervical adenocarcinoma patients.

The limitations of this study included the absence of more detailed patient information including pathological features, radiologic findings, and laboratory indicators. Further studies including a large series with comprehensive information and detailed survival data would be needed. Nevertheless, the extension of our new system to an online program that can update with new measures should be expected.

## Conclusion

In this paper, we developed a deep neural network model for cervical adenocarcinoma patients using data from the SEER database. The performance of this model was shown to be superior to other survival prediction models including the CPH model, LMT model, RSF model, and SVM model in the test set. Real-word information on cervical adenocarcinoma patients was also incorporated to validate the DL model. The results of external validation supported the possibility that the model can be used in clinical work. Finally, new survival grouping and personalized prediction systems were proposed which provided more accurate prognostic information for patients.

## Supplementary Information

**Additional file 1: **Details of 2501 patients we selected in the SEER database.

**Additional file 2:** Details of 220 patients in the external testing set.

## Data Availability

Publicly available datasets were analyzed in this study. This data can be found here: https://seer.cancer.gov/data/. Other data generated or analyzed during this study are included in this published article and its supplementary information files. Details of 2501 patients we selected in the SEER database were showed in “SEER-adenocarcinoma of cervix” file. Cases in the external-test set were retrospectively collected at Qilu Hospital Shandong, China. Details of 220 patients in the external testing set were showed in “adenocarcinoma of cervix in Qilu hospital.” file.

## Abbreviations

DL: Deep learning
NCCN: The National Comprehensive Cancer Network
SCC: Squamous cell carcinoma
AC: Adenocarcinoma
FIGO: The International Federation of Gynecology and Obstetrics
OS: Overall survival
CPH: Cox proportional hazards
K–M: Kaplan–Meier
LMT: Linear multi-task
RSF: Random survival forest
SVM: Support vector machine
SEER: Surveillance, epidemiology and end results
IRB: The institutional review board
TH: Hysterectomy
LND: Hysterectomy and lymph node dissection
BSO: Bilateral salpingo-oophorectomy
N-MTLR: Neural multitask logistic regression
IBS: Integrated Brier scores
ROC: Receiver operating characteristic
AUC: Area under the curve
AE: Absolute error
